# Non-steroidal anti-inflammatory drugs reduce pleural adhesion in human: evidence from redo surgery

**DOI:** 10.1038/s41598-023-41680-7

**Published:** 2023-09-04

**Authors:** Peter Sze-Yuen Yu, Kin-Wai Chan, Chiu-On Tsui, Shun Chan, Kin-Hoi Thung

**Affiliations:** 1 Division of Cardiothoracic Surgery, Department of Surgery, Tuen Mun Hospital, Hong Kong, Hong Kong; 2 Department of Statistics, The Chinese University of Hong Kong, Hong Kong, Hong Kong

**Keywords:** Inflammation, Surgery

## Abstract

Non-steroidal anti-inflammatory drugs (NSAIDs) reduced pleural adhesion in animal studies, but its effect on human had not been studied. A retrospective study was carried out for patients with solitary pulmonary nodules without a pre-operative tissue diagnosis positive for malignancy. The impact of the use of NSAIDs after stage one wedge resection was assessed by the degree of pleural adhesions encountered during second-stage, redo completion lobectomy. From April 2016 to March 2022, 50 consecutive patients meeting the inclusion criteria were included, and 44 patients were selected for analysis after exclusion (Treatment group with NSAID: N = 27; Control group without NSAID: N = 17). The preoperative characteristics and the final tumor pathologies were similar between the groups. The use of NSAID was significantly associated with lower risk of severe pleural adhesions and complete pleural symphysis (risk difference = −29%, *p* = 0.03). After controlling the effect of tumor size and chest drain duration, only the use of NSAID was statistically associated with the lowered risk of severe pleural adhesions and complete pleural symphysis. No statistically significant effects of NSAID on operative time (*p* = 0.86), blood loss (*p* = 0.72), and post-operative length of stay (*p* = 0.72) were demonstrated. In human, NSAIDs attenuated the formation of pleural adhesions after pleural disruptions. Physicians and surgeons should avoid the use of NSAIDs when pleural adhesion formation is the intended treatment outcome.

Non-steroidal anti-inflammatory drugs (NSAID) are commonly prescribed for pain control. Its anti-inflammatory effect can theoretically reduce pleural inflammation, fibrosis, and subsequent pleural adhesion. The study on the effect of anti-inflammatory drugs on pleural adhesions were limited to animal models. Those were consistent results demonstrating that the degree of pleural adhesions was decreased after the use of Aspirin^1^, Diclofenac^2–4^ and Prednisolone^4^. Certain retrospective studies in human failed to demonstrate a significant adverse impact of the use of NSAIDs on the recurrence rate after pleurodesis for spontaneous pneumothorax^5–7^; however, those clinical studies did not provide any macroscopic or microscopic comparison of the different quality and extent of pleural adhesion formation. Despite the conflicting results, surgeons tend to call off the routine use of NSAID after mechanical pleurodesis to avoid attenuation of the quality of pleural symphysis. To the best of our knowledge, there have been no human studies on the impact of anti-inflammatory drugs on pleural adhesions after pleural disturbances. It is not without significant ethical challenges to conduct any prospective trials on human, especially patients after chemical or surgical pleurodesis, on the effect of NSAID with this known risk in animal studies.

Surgical pleural disruptions lead to formation of pleural adhesions secondary to pleural inflammation. Severe pleural adhesion increases the risk of conversion from VATS to thoracotomy, and postoperative surgical complications^8,9^. Redo thoracic surgery are commonly performed in patients with recurrent lung cancers, or staged completion lobectomy after previous wedge biopsy of lung tumours. This could offer surrogate evidence to the effect of NSAID on pleural adhesions after pleural disruption, because the severity of pleural adhesions could be well seen and assessed during the second operation.

From evidence available from animal studies, it was hypothesized that the use of NSAIDs in human also attenuates pleural adhesions after VATS. This study aimed to investigate the effect of NSAIDs after first-time lung operation on the risk of severe pleural adhesions and complete pleural symphysis, which was assessed during stage-two redo completion lobectomy.

## Methods

### Operative strategy

Patients with solitary pulmonary nodule without a definite pre-operative tissue diagnosis were subjected to direct surgery. The operations were performed by the same team of two experienced specialist surgeons. Trainees were only allowed to perform the operations under close supervisions from the experienced specialist surgeons. Under general anesthesia with double-lumen endotracheal intubation, patients were placed in full lateral decubitus position. Standard utility port was created at the 4th, 5th or 6th intercostal space, and an additional camera port may be placed at the 7th or 8th intercostal space on the mid-axillary line. Wedge resection of lung including the target nodule was performed using endostaplers. Pleural cavity was routinely irrigated with at least 2 litres of water. One Fr 24 or 28 chest drain was placed, and the utility wounds were closed in the routine manner. Negative drain suction (-15 cmH2O) was added until at least post-operative day 1. Pain control during early postoperative days was routinely achieved by oral paracetamol and tramadol or dihydrocodeine, with or without intravenous patient-controlled analgesia. NSAIDs (Cyclooxygenase-2 inhibitors e.g. Celecoxib or Etoricoxib) may be prescribed at the discretion by the pain specialists. Patients were discharged from hospital after chest drain removal. Patient with invasive primary lung malignancy confirmed on formal paraffin section, except those with pure lepidic adenocarcinoma, received the second stage redo surgery. Any pleural adhesions encountered were taken down with diathermy. Completion lobectomy was performed using individual ligation technique for bronchovascular structures, followed by mediastinal lymph node sampling or dissection according to the latest revised European Society of Thoracic Surgeons guidelines.

### Patient and data

**Figure 1:**
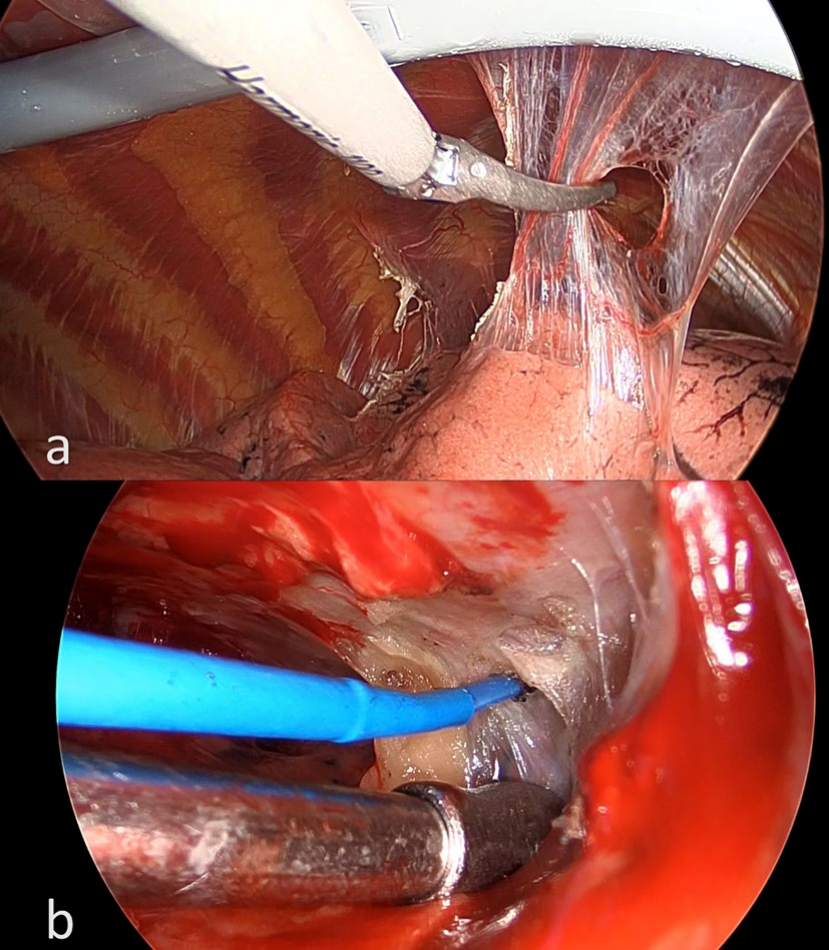
Intraoperative view of the pleural cavity showing mild amount of pleural adhesions (**a**) and complete pleural symphysis (**b**).

The study was performed in accordance with the Declaration of Helsinki, and in accordance with the relevant guidelines and regulations. The medical records of consecutive patients were retrieved from patients’ case notes and electronic medical records. Data collected included preoperative patient demographic and comorbidities, disease status, operative procedures, and postoperative outcomes. Patients who met the following criteria were included in the study: (1) solitary lung nodule with no definite pre-operative tissue diagnosis; (2) underwent VATS wedge resection only in the stage one operation; and (3) confirmed primary lung malignancy requiring second-stage completion lobectomy. Patients with the following characteristics were excluded because they were apparent and significant confounders affecting the formation of pleural adhesions: (1) any mediastinitis or pleural space infection before or after the stage one operation; (2) presence of dense pleural adhesions or complete pleural symphysis in the stage one operation; (3) hemothorax or required chemical pleurodesis after the stage one operation; (4) use of Aspirin or glucocorticoids before or after the stage one operation. The primary outcome was severe pleural adhesions and complete pleural symphysis encountered during the stage two redo operation (Fig. 1). Secondary outcomes included operative time, blood loss, persistent air leak and post-op length of stay after the stage two operation. Severe pleural adhesion was defined as dense pleural adhesions involving two or more pleural surfaces (anterior, lateral, posterior, mediastinal, diaphragmatic), and complete pleural symphysis was defined as the complete lack of tissue plane between the parietal and visceral pleura in the majority of pleural surfaces.

### Statistical analysis

Descriptive statistics were used to compare the variables between the groups. Continuous variables were reported as mean value (± standard deviation) and compared using the two-sided t-test if they conform with normal distribution (verified by Normal Probability Plot Test); otherwise they were reported as median value and interquartile range, and Mann–Whitney U test would be employed. Categorical variables were reported as counts and percentages, and differences between two groups were assessed by Chi-square test, or two-sided Fisher’s exact test if a cell value was lower than 5. All statistics were analysed using the SPSS for Windows version 26.0 (SPSS Inc., Chicago, IL, USA) or R (Version 4.1.0). A *p*-value < 0.05 represents statistically significant difference. Both univariate and multivariate analyses were performed to analyse the association of NSAID and the outcome variables, which include occurrence of severe pleural adhesions and complete pleural symphysis, length of operative time, amount of blood loss, and post-operative length of stay. For the univariate analysis, the one-sided Boschloo’s test^10^ and one-sided t-test were used for analysing categorical and continuous variables, respectively. For multivariate analysis, a linear regression model and a probit regression model were used to model the association pattern for categorical and continuous variables, respectively. All models were fitted after controlling the effects of duration of chest drain and tumour size. Missing data entries are multiply imputed 100 times by chained equations using the R package “mice” upon assuming that it was missing at random^11,12^. The multiply imputed results were pooled by using the formulas in Chan and Meng^13^ and Chan^14^.

### Ethical approval and consent to participate

The ethical approval was granted by the Research Ethics Committee, New Territories West Cluster, Hospital Authority, Hong Kong SAR (Approval number: NTWC/REC/22019). Informed consent was waived due to retrospective nature of the study.

## Results

From April 2016 to March 2022, 389 pulmonary wedge resections were performed. A total of 50 consecutive patients fulfilling our inclusion criteria were included, and 44 patients were selected for further analysis (6 patients had concomitant use of Aspirin or glucocorticoids before and/or after the first operation and were excluded) and divided into 2 groups: 27 patients who received NSAID after stage one operation, and 17 patients who did not receive NSAID. The two cohorts had similar perioperative and intraoperative characteristics (Table 1). There were 19 patients (43.2%) who had history of malignancies. Moreover, the two groups also had similar pathological types and T stage of the primary lung tumours (Table 2). Of note, only 1 out of 44 patients had indeterminate origin of the malignant lung tumor even by formal paraffin section. There were 5 patients in each group who had history of oncological treatments. Majority of the stage one operations were uniportal VATS. During stage two redo completion lobectomy, no conversion from VATS to thoracotomy was required for all patients.

**Table 1:** Perioperative and intraoperative parameters of the two study cohorts after the stage one lung wedge resection. Continuous variables were expressed in mean $\pm$ standard deviation. For each clinical characteristics shown in the above table, no significant difference was found among the control group and treatment group at a 5% significance level. It showed covariate balance between control and treatment groups.

Clinical characteristic	Treatment, with NSAID^a^ (n = 27)	Control, without NSAID (n = 17)	*p*-Value (2-sided test)
Age	61.7 ± 6.8	62.1 ± 7.5	0.88
Smoker	6 (22.2%)	7 (41.2%)	0.32
Diabetic	5 (18.5%)	5 (29.4%)	0.64
Hypertension	8 (29.6%)	7 (41.2%)	0.65
Asthma	0 (0%)	1 (5.9%)	0.39
History of Malignancy	9 (33.3%)	10 (58.8%)	0.18
History of Oncological therapy	5 (18.5%)	5 (29.4%)	0.40
Forced expiratory volume in one second (% predicted)	96.9 ± 14.9	87 ± 19.9	0.07
Diffusing capacity for carbon monoxide (% predicted)	99.2 ± 15.9	89.7 ± 20	0.09
Upper lobe resection	14 (51.9%)	8 (47.1%)	1.00
Uniportal VATS^b^	21 (77.8%)	14 (82.4%)	1.00
Right-side operation	21 (77.8%)	10 (58.8%)	0.32
Visceral pleural sealants	7 (25.9%)	3 (17.6%)	0.72
Smaller chest drain size (Fr 24)	21 (77.8%)	14 (82.4%)	1.00
Chest drain duration (days)	4.1 ± 2.5	4.6 ± 4.1	0.63
Length of stay (days)	5.8 ± 2.9	6.4 ± 3.2	0.55
Waiting time for redo operation (days)	104.8 ± 85.8	106 ± 73.9	0.96

^a^NSAID: Non-steroidal anti-inflammatory drugs.

^b^VATS: Video-assisted thoracic surgery.

**Table 2:** The pathological types of the resected primary tumor and their T stages. No significant difference was found among the control group and treatment group at a 5% significance level. It showed covariate balance between control and treatment groups.

Clinical characteristic	Treatment, with NSAID^a^ (n = 27)	Control, without NSAID (n = 17)	*p*-Value
Tumor pathology			
Adenocarcinoma	26 (96.3%)	13 (76.5%)	0.07
Squamous cell carcinoma	0 (0%)	2 (11.8%)	0.14
Lymphoepithelioma-like carcinoma	1 (3.7%)	0 (0%)	1.00
Poorly differentiated carcinoma	0 (0%)	1 (5.9%)	0.39
Carcinoma, origin indeterminate	0 (0%)	1 (5.9%)	0.39
T stage			
pT1a	8 (29.6%)	5 (29.4%)	1.00
pT1b	11 (40.7%)	6 (35.3%)	0.97
pT1c	5 (18.5%)	4 (23.5%)	0.72
pT2b	2 (7.4%)	1 (5.9%)	1.00
pT3	0 (0%)	1 (5.9%)	0.39
pTmi	1 (5.3%)	0 (0%)	1.00

^a^NSAID: Non-steroidal anti-inflammatory drugs.

First, we reported the mean for each dependent variables within each treatment group. The 95% confidence intervals of the means were also provided (Table 3). We conjectured that the use of NSAID is associated with lower risk of severe pleural adhesions and complete pleural symphysis, longer operative time, more blood loss, higher incidence of persistent air leak and longer post-operative length of stay. The one-sided Boschloo's test was used to analyze the association between the usage of NSAID and pleural adhesions. Note that Boschloo's test has been proven to be uniformly more powerful than the classical Fisher’s exact test at a cost of longer computation time. The one-sided t-test was used to analyze the association between the usage of NSAID and each of the remaining three continuous dependent variables. Overall, the use of NSAID was statistically associated with lower risk of severe pleural adhesions and complete pleural symphysis (risk difference =  − 29%, *p* = 0.03); however, it had no statistically significant effect on operative time, blood loss, post-operative persistent air leak and post-operative length of stay.

**Table 3:** Table showing the operative outcomes after stage two redo completion lobectomy. The use of NSAID was significantly associated with reduced risk of severe pleural adhesions and complete pleural symphysis, but not operative time, blood loss, persistent air leak and post-operative length of stay. For pleural adhesions, the result was presented in terms of absolute risk and the 95% confidence intervals. p-value was obtained from the one-sided Boschloo's test. (Note: the primary outcome “severe pleural adhesions and complete pleural symphysis” were denoted as “pleural adhesions” in the table for simplicity).

	Treatment: with NSAID^a^ (N = 27)	Control: without NSAID (N = 17)	*p*-Value
Pleural adhesions (absolute risk and 95% confidence intervals)	0.19 (0.04–0.33)	0.47 (0.23–0.72)	0.03
Operative time (minute)	157 ± 21	173 ± 21	0.86
Blood loss (mL)	131 ± 50	152 ± 15	0.72
Persistent air leak	7 (25.9%)	7 (41.2%)	0.15
Post-op length of stay (day)	5.8 ± 1.1	6.4 ± 1.5	0.72

^a^NSAID: Non-steroidal anti-inflammatory drugs.

Second, we performed more detailed analysis by controlling the effect from potential confounding factors, including duration of chest drain and tumor size (which was a surrogate marker to reflect the volume of lung parenchyma resected). Probit regression model was used for fitting the binary dependent variable (i.e. pleural adhesions), whereas a linear regression models were used for fitting the continuous dependent variables (i.e. operative time, blood loss, and post-operative length of stay) (Table 4). After controlling the effect of duration of chest drain and tumor size, the use of NSAID was the only parameter significantly associated with the risk of severe pleural adhesions and complete pleural symphysis (*p* = 0.03). Given mean duration of chest drain and mean tumor size, the probability of severe pleural adhesions and complete pleural symphysis decreased from 49% to 16% if NSAID was used.

**Table 4:** Probit regression analysis for the binary dependent variable (i.e. pleural adhesions), and linear regression analysis for the continuous dependent variables (i.e. operative time, blood loss, and post-operative length of stay). After controlling for the effect of duration of chest drain and tumor size, the use of NSAID was significantly associated with reduced risk of severe pleural adhesions and complete pleural symphysis, but not operative time, blood loss or post operative length of stay. (Note: the primary outcome “severe pleural adhesions and complete pleural symphysis” were denoted as “pleural adhesions” in the table for simplicity). Estimate: regression coefficient estimate; NSAID: Non-steroidal anti-inflammatory drugs.

Effect	Estimate	Standard error	*p*-Value (2-sided test)
Pleural adhesions			
(Intercept)	− 0.502	0.590	0.39
NSAID	− 0.957	0.435	0.028
Duration of chest drain	− 0.0353	0.0677	0.60
Tumor size	0.0388	0.0254	0.13
Operative time (minute)			
(Intercept)	158	21.8	< 0.001
NSAID	− 20.3	15.4	0.20
Duration of chest drain	− 2.04	2.40	0.40
Tumor size	1.62	0.914	0.08
Blood loss (mL)			
(Intercept)	83.3	54.9	0.14
NSAID	− 21.7	39.3	0.58
Duration of chest drain	2.85	5.99	0.64
Tumor size	3.43	2.78	0.14
Post-op length of stay (day)			
(Intercept)	2.70	1.14	0.02
NSAID	− 0.484	0.807	0.55
Duration of chest drain	0.481	0.125	< 0.001
Tumor size	0.0938	0.0478	0.057

## Discussion

Pre-operative tissue diagnosis is indicated for solitary lung nodules before subjecting patients to major lung resections under general anaesthesia. This is however limited by the diagnostic yield of tissue sampling modalities. The yield from bronchoscopy under fluoroscopic guidance was 53–82%^15^. The yield from CT-guided percutaneous biopsy was 84–97%, with particular concerns for small nodules^16^, and nodules adjacent to pericardium and great vessels^17^. Therefore, certain patients need to receive wedge biopsy of lung without a definite pre-operative tissue diagnosis. Moreover, there was also low concordance between biopsy and surgical pathology in determining the predominant histological subtype of primary lung adenocarcinoma^18^, certain of which may be cured by simple, non-anatomical wedge resection without the need of anatomical lobectomy^19^. Intra-operative frozen section is time-consuming and has limited concordance with the final histological subtype on paraffin section. Differentiation between primary and metastatic lung adenocarcinoma is also better by formal histology and marker identification than frozen section. Indeed, staged completion lobectomy is not favoured by thoracic surgeons due to the formation of diffuse pleural adhesions after stage one operation, and the need of painstaking efforts to perform adhesiolysis during redo operation which complicated increased operative time, blood loss, post-operative air leak and length of hospital stay^20^. NSAIDs are known effective oral analgesics for post-thoracotomy pain with good safety profile in general^21,22^. Association of NSAIDs with weakening of post-inflammatory pleural adhesions could be regarded as a double-edged sword. To take its advantages, surgeons can consider generous prescription of post-operative NSAIDs when staged redo surgery is highly expected. To avoid its disadvantages, physicians and surgeons should avoid prescription of peri-procedural NSAIDs when pleurodesis is intended.

Formation of pleural adhesions relied primarily on the occurrence of inflammatory processes. In an animal model, NSAIDs were shown to attenuate inflammatory process reflected by the reduction of pleural exudation, neutrophil count, transforming growth factor-β etc. Moreover, NSAIDs also increased the intensity of fibrinolysis process shown by the increase in D-dimers and tissue plasminogen activator^2^. Conflicting results were seen in another study which microscopically examined the quality of pleurodesis after silver nitrate or talc pleurodesis with or without corticosteroids or NSAIDs. The effect of steroids and NSAIDs on pleural adhesions were not apparent after silver nitrate pleurodesis but talc pleurodesis^4^. Thus, the anti-fibrosis and pro-fibrinolytic effect of NSAIDs may be a significant factor, but not a solely sufficient factor, for the quality of pleurodesis. It also depends on the strength of the stimulant of the pro-inflammatory process (e.g. surgical trauma, chemical stimulation). Surgical trauma may not be the strongest pro-fibrosis stimulant of all, but in a porcine model the use of NSAIDs was associated with the reduction of quality of pleurodesis by both macroscopic and microscopic examination^3^. A number of retrospective studies investigated the clinical impact of NSAIDs on the recurrence rate after pleurodesis for spontaneous pneumothorax in human^5–7^. Despite the results showing lack of association between the use of NSAIDs and recurrence of pneumothorax, it has to be reminded that: ^1^ those clinical studies had not provided visualized, macroscopic evidence of the degree of pleural adhesions; and ^2^ the potency of anti-inflammatory effect (such as Ketorolac, as in those studies) among different types of NSAIDs are highly variable. The use of cyclooxygenase-2 inhibitors in our study might have resulted in different degree of attenuation of adhesion formation compared to Ketorolac, Diclofenac or Aspirin in other studies. Combining the available evidence from previous studies and our current clinical study on human participants, we advocate the judicious use of perioperative NSAIDs according to whether formation of pleural adhesions was the desired clinical outcome. Particular cautions should be exercised for perioperative pain control for patients after pleurodesis when other reasonable alternative analgesics, with no pharmacological mechanisms related to the inflammatory or fibrinolytic processes, were available.

The first major limitation of this study is the small sample size. The recruited patients represent the minority of the routine surgical population. Stringent inclusion and exclusion criteria with the intention of improving the homogeneity of the cohorts lead to further truncation of the number of study cases. However, ensuring homogeneity is more important than solely maximizing sample size without addressing significant confounders. Moreover, small sample size does not negate the association between use of NSAIDs and attenuation of pleural adhesions, as long as the correct statistical analyses are performed. Fisher's exact test is well-known to have low power and needs restrictive assumptions (such as fixed column sums and fixed row sums). Some more powerful tests have been proposed in the literature. One of the best methods so far is Boschloo's test, despite its much longer computation time even for a small sample size. The second major limitation of this study is the retrospective nature, which inherently carries selection bias and non-standardized definition of parameters and perioperative management. The completeness of lung expansion after wedge resection, which would affect the formation of adhesions, were also affected by the size and volume proportion of the lung wedge taken, patient’s pain level and use of incentive spirometer, and the amount of pneumothorax after chest drain removal. Thirdly, the definition of ‘severe’ pleural adhesions was from the surgeons’ subjective impression without quantitative measurements, which were surgically not possible in human. In this single-centre study, the definition of severity of pleural adhesions is uniform across the operating surgeons. Still, it is intrinsically difficult to quantify the density, focality and the proportional surface area of pleural surface involved by adhesions. To reflect the operative difficulty of the redo operations, multiple parameters (operative time, blood loss, postop length of stay) were used. Nevertheless, each of these parameters were also affected by a number of other operative factors rather than solely by the degree of pleural adhesions. Fourthly, limited by retrospectice nature of the study, the compliance, frequency of use (regular vs. as-required basis), and the total intake dosage of NSAIDs could not be accurately measured or recalled. The criteria of NSAIDs prescription, which was from the clinical discretion by pain specialists, were also not standardized. Fifthly, routine monitoring of post-operative inflammatory markers or cytokines were not performed. The variation of levels of these markers, independent of the effect of NSAIDs, may correlate with different severity of pleural adhesions after pleural injury. These limitations can only be addressed better in randomized and controlled trial settings, with sophisticated measurement of the severity of pleural adhesions. Last but not least, our study lacked microscopic examination of the quality of pleural adhesions. This requires sharp excision of the parietal pleura, together with the adhesion bands and a wedge of healthy lung tissue. This manipulation in human participants is difficult to justify ethically because it, beyond any clinical indications, increases the risks of bleeding and air leak.

## Conclusion

We present the first human study demonstrating the effect of NSAIDs on attenuating formation of pleural adhesions. After the first stage lung wedge resections, routine use of NSAIDs may facilitate subsequent second stage redo completion lobectomy by reducing the risk of severe pleural adhesions and complete pleural symphysis. Physicians should be cautious with the use of NSAIDs as analgesics when pleurodesis is the desired major clinical outcome.

## Data Availability

The datasets generated and/or analysed during the current study are not publicly available by default due to restrictions from institutional ethics review board, but are available from the corresponding author on reasonable request.
